# Editorial: Tooth enamel research: Enamel 10 and beyond

**DOI:** 10.3389/fphys.2023.1323504

**Published:** 2023-11-07

**Authors:** Pamela K. Den Besten, Thomas G. H. Diekwisch

**Affiliations:** ^1^ The Department of Orofacial Sciences at the University of California at San Francisco, San Francisco, CA, United States; ^2^ Eastman Institute for Oral Health, The University of Rochester, Rochester, NY, United States

**Keywords:** enamel, amelogenesis, biomineralization, amelogene, ion transport

Enamel 10—enamel in transition. Enamel 10 will forever be remembered as the Pandemic era enamel conference. Even though the number of COVID-19 infections was already declining by the time the meeting was finally held (May 8–11), some of the participants were still wearing masks, and social distancing, a term unheard of at any of the previous enamel meetings, was still encouraged. As part of pandemic era precautions, the meeting had to be postponed and no longer occurred at the Oglebay Research and Conference Center in Wheeling, West Virginia, but instead was held at the University of Pittsburgh, breaking with the long tradition of enamel meetings convened at locations remote from universities. And yet, the meeting still drew a remarkable number of more than 75 attendees fiercely committed to uphold the traditions of these legendary Enamel meetings giving testimony to the unbreakable spirit of conference participants and continued progress of enamel research even during a time of adversity. The papers in this Frontiers Research Topic, along with the abstracts of the presentations that can be viewed in the supplemental material, represent the state of the science of enamel research.

In the final discussion session of the meeting participants, there were reflections as to the beauty and complexity of the enamel structure, and the great work ahead to fully understand enamel structure and the biology of the cells and proteins that result in the synthesis of this unique tissue. The International Enamel symposiums, spanning from the first meeting in London in 1964 to the present, hold a history of research scientist who have made enormous contributions to the science of the field of enamel research. The proceedings of this Enamel 10 meeting includes a commemoration to one of the early heroes of enamel research, Dr. Alan G. Fincham, an early pioneer in amelogenin biochemistry and self-assembly research (Diekwisch).

In their discussions, the participants reflected on the complex systems that join together to form fully mineralized enamel, and the need for continued collaboration from scientists from different fields of research. Animal models hold great promise in understanding how individual genes, many of which were not known to have key roles in enamel formation. Characterization of these animal models requires collaboration or people sharing expertise, approaches and knowledge ranging from stem cells to structural biology. Along with *in vivo* models is the continued need to develop relevant *in vitro* models, to more directly assess cellular mechanisms. The group reflected on the translational implications of enamel research, including how enamel defects represent biomarkers for other systemic effect diseases; both environmental and genetic. The group encouraged future studies with broad minded scientific approaches, including new opportunities in data science, and collaborations with scientists working in other organ systems.

The following is a list of categories of presentations, and the papers in each category. Further information on work that was presented at the Enamel 10 meeting can be viewed in the abstracts, that are included as a supplement to this Research Topic.

Enamel pathologies. Since the earliest years of anatomical studies, disease has been the teacher of developmental and structural insight. Two unique enamel pathologies have a critical role in health and wellbeing: molar hypoplasia and hereditary Amelogenesis imperfecta type enamel defects. Both of these conditions result in the need for extensive dental restorative treatment and frequently lead to tooth loss beginning in childhood. Molar hypoplasia, which is associated with trapped serum albumins in the tooth enamel of first permanent molars (Hubbard et al., 2021), likely due to early childhood or material events leading to hypomineralization, is highly associated with caries (Yardimci et al., 2021). In the present volume, Zhang et al. have used a machine learning approach to report on the protective association between dental fluorosis and molar hypomineralization (Zhang et al.).

Amelogenensis Imperfecta (AI) is a collection of genetic defects that result in poorly formed enamel. The Reference Centre for Rare Oral and Dental Diseases, has undertaken a re-classification of genetic data from 111 families and employed 567 genes based on their Next-generation sequencing (NGS) panel GenoDENT. This NGS study has emerged as a cost-effective approach to define the genetic basis of amelogenesis imperfecta (Rey et al., 2019; Bloch-Zupan et al.).

Amelogenin structural biology and histidines. Amelogenin structural biology has contributed greatly to our understanding of enamel development and protein/mineral interactions. While in the past, much attention has been paid to the elongated polyproline stretches at the C-terminus and the N-terminal alpha helix, this time around, the histidine-rich amelogenin stretches finally came to shine. Histidine is one of four amino acids uniquely elevated in amelogenins (Eastoe, 1963) and known for its buffering qualities and pH-dependent flexibility. In the present volume, investigators have studied the effect of histidine residues on adsorption interactions between amelogenin and apatites during mineralization (Tao et al.) and in self-assembly of the insoluble amelogenin self-assembly domain (Zhang et al.). Another study on the role of calcium on amelogenin self-assembly provided much insight into the role of calcium ions during amelogenin self-assembly (Zhang et al.; Zhang et al.).

**FIGURE 1:**
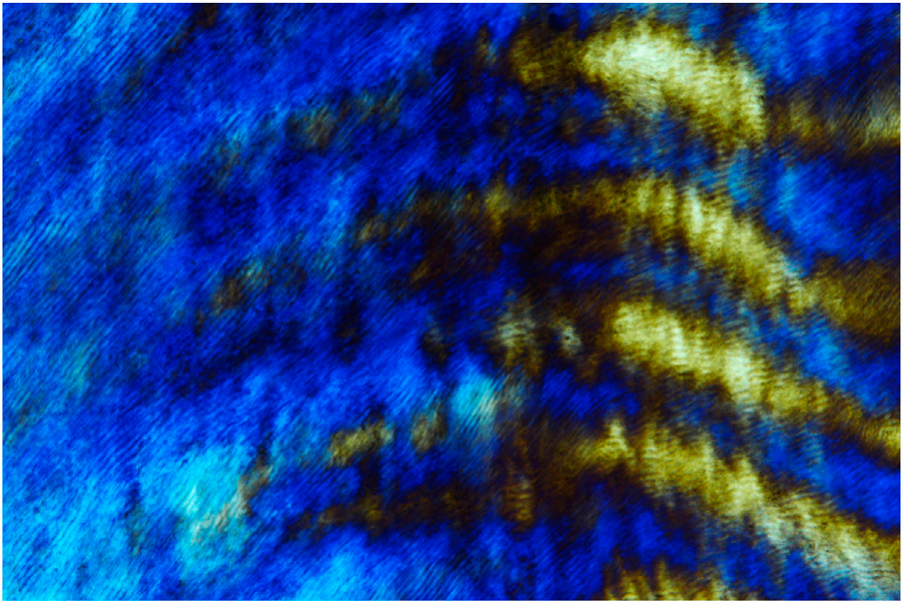
Polarization microscopic image of human enamel prisms.

Enamel rod formation. The unique perpendicular orientation of mammalian enamel rods is one of the hallmarks of vertebrate enamel structure (Figure 1). On a cellular level, the secretory pole of ameloblasts faces the growth end of elongating enamel rods. Relative ameloblast movement away from the secretory aspect of the elongating enamel rod facilitates the formation of the characteristic Herringbone pattern that contributes to enamel mechanical stability. Most recently, two molecules have been identified that may play a significant role in the formation of enamel prisms: i) ADAM10 and ii) the ameloblastin amphipathic helix. ADAM10 is a Disintegrin And Metalloproteinase (ADAM) family member and sheddase that cleaves anchored cell surface proteins (Shahid et al.). ADAM10 may also promote cell movement through its interactions with tetraspannins, lending credibility to a potential role in prism formation and decoupling between ameloblasts and growing prism surfaces. The discovery of ADAM10 function in prism elongation coincided with another key discovery related to enamel prism growth, the role of the Ameloblastin amphipathic helix in the regulation of several cell polarization genes, including Vangl1, Vangl2, Prickl1, ROCK1, ROCK2 and Par3 (Su et al., 2022). As a result, the amphipathic ameloblastin matrix may play a role in facilitating cell membrane interactions and ameloblast cell polarization as the onset of enamel prism formation (Su et al., 2022).

Ion transport. One of the most intriguing questions in enamel research has been the question how massive amounts of calcium, phosphate and other ions are transported from the blood vessels into the mineralizing enamel layer. Pioneering studies by Lacruz and coworkers (Lacruz et al., 2017) have established the role of calcium stores (SOCE) and Ca^2+^ release activating Ca^2+^ (CRAC) channels in the control of these channels and processes by stromal interaction molecules 1 & 2 (STIM1 and 2), and the plasma membrane pore subunit of the CRAC channel ORAL1 (Lacruz et al., 2017; Said et al.). Studies in this volume (Said et al.) have demonstrated that loss of STIM1 results in a hypomineralized enamel phenotype and misregulation of several calcium transport-associated genes. Not only calcium and phosphate, but also other ions such as sodium and potassium contribute to the mineral phase of tooth enamel. New reports at the Enamel 10 symposium suggest that potassium transport is regulated by Kir4.2 in maturation ameloblasts and sodium uptake is regulated by the Na^+^/Ca^2+^ exchanger Nckx4 (Ngu et al.). *In vitro* evidence suggests that K+ and Na2x uptake may be regulated through WDR72-mediated pH dependent endocytosis and membrane trafficking, another line of evidence for the importance of pH in amelogenesis (Ngu et al.).

Non-SCPP proteins in the enamel organ. Previous enamel meetings prominently featured temporo-spatial proteomics studies (Pandya et al., 2017) or the spatial distribution and function of non-amelogenin SCPP proteins (Diekwisch, 2011). In contrast, the current proceedings highlighted several of the proteins in the enamel matrix previously not linked to amelogenins or SCPP proteins, including STIM1 (Said et al.) and Keratin 75 (Deshmukh et al.). These and another study related to Keratin 14 (Lu et al., 2011) pay homage to the role of keratins as epithelial structural proteins in the enamel organ as an epithelial tissue. Together, these five paragraphs feature some of the trends and highlights of Enamel X. We apologize in advance to those authors who have contributed excellent papers but whom we have not mentioned in this overview due to limitations of space.

The future: Enamel in Paris. The next enamel meeting, Enamel XI, will be organized by Drs. Sylvie Babajko and Catherine Chaussain in Paris in 2026, once more gathering enamel scientists from all over the world. It is anticipated that the current trends in enamel research will continue, especially in the areas of environmentally caused enamel defects, ion transport and enamel prism growth. Matching trends in other disciplines, such as single cell-based studies, will shed light on the contribution of individual enamel organ cells toward amelogenesis; and tissue engineering approaches will have become further advanced in terms of biological and synthetic enamel engineering. Regardless of the individual area of research, chances are that young and old enamel researchers will stroll on the shores of the river Seine and walk along the Tuileries to discuss questions about enamel research. Enamel research will have made much progress due to the multitude of models and resources available through the EnamelBase consortium (https://www.facebase.org/resources/enamelbase/), and results from studies involving all of these intriguing new model systems will be shared in the city of eternal Gothic architecture somewhere between Sainte Chapelle and Notre Dame (reconstruction anticipated in April 2024).
